# 303. Tissue-compartment specific differences in T-cell response found in 44 fatal COVID-19 cases

**DOI:** 10.1093/ofid/ofad500.375

**Published:** 2023-11-27

**Authors:** Trevor M Stantliff, Andrew Platt, Syndey R Stein, Cihan Oguz, Kevin M Vannella, Sabrina Ramelli, Stephen Hewitt, Daniel S Chertow

**Affiliations:** University of Cincinnati, Cincinnati, Ohio; National Institute of Allergy and Infectious Diseases, Washington, DC; Clinical Center, NIH, Bethesda, Maryland; National Institutes of Health, Bethesda, Maryland; Clinical Center, NIH, Bethesda, Maryland; National Cancer Institute, Bethesda, Maryland; National Cancer Institute., Bethesda, Maryland; Clinical Center, National Institutes of Health, Bethesda, Maryland

## Abstract

**Background:**

T-cells play an essential role in recognizing and clearing viruses from infected tissues. While the peripheral blood T-cell responses among patients with coronavirus disease 2019 (COVID-19) has been well characterized in the recent literature, little is known about the overall repertoire diversity of T-cell responses and SARS-CoV-2-specific T-cell breadth and depth across tissues in patients with severe COVID-19. To fill this knowledge gap, we evaluated the T-cell immune repertoires using bulk sequencing of multiple tissue samples from a cohort of fatal COVID-19 cases.

**Methods:**

Lungs, thoracic lymph nodes and spleens were collected at autopsy from a cohort of 44 patients who died with or from COVID-19. DNA was isolated from paraffin embedded tissues fixed with an ethanol buffer and from peripheral blood mononuclear cells (PBMCs) collected perimortem. T-cell receptor beta (TCRb) sequencing was performed using the Adaptive ImmunoSeq platform and the SARS-CoV-2-specific T-cell response was quantified by exact matching of the TCRb sequences with those reported in the ImmuneCODE and VDJdb databases.

**Results:**

Significantly lower T-cell clonality and higher numbers of unique T-cell rearrangements were observed in lymph nodes compared to the lung tissues and PBMCs across all patients, indicating higher T-cell diversity in the lymph nodes. Clonal expansion was greatest in the PBMCs and least in the lymph node tissues. Pearson correlations between age and SARS-CoV-2 specific T-cell depth, which were controlled for gender and the number of days between onset and death, varied significantly across tissue compartments in both magnitude and directionality. We observed a strong positive correlation (0.35, *p*< 0.05) between the SARS-CoV-2 specific T-cell depth and older age in the lymph node samples, that was in contrast with an opposite trend in the PBMCs (-0.52, *p*< 0.001).

**Figure 1:**
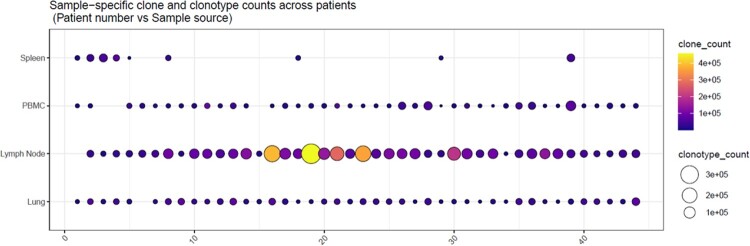
Lymph node tissues had increased T-cell counts (clone_count: color scale) and increased unique T-cells (clonotype_count: circle size) when compared to PBMCs and lung tissues.

**Figure 2.**
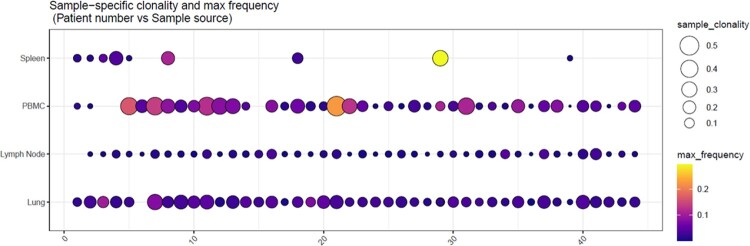
PBMC and lung samples have increased clonality (sample_clonality: circle size) and max frequency for the immunodominant clonotype (max_frequency: color scale) when compared to lymph node tissues.

**Conclusion:**

The higher depth of SARS-CoV-2 specific T-cells within lymph nodes of older patients that was not observed within circulating PBMCs suggest a deficit of translocation of virus-specific T cells from the site of antigen presentation to circulation and the site of infection, with implications for the increased severity of disease observed in older patients with COVID-19.

**Disclosures:**

**All Authors**: No reported disclosures

